# Establishment of a method to determine the magnetic particles in mouse tissues

**DOI:** 10.1186/1556-276X-7-665

**Published:** 2012-12-06

**Authors:** Yifan Wu, Wuxu Zhang, Yuxia Wang, Qian Li, Guo Gao, Na Dong, Hengyao Hu, Kan Wang, Junhua Wu, Zhongcai Gao, Daxiang Cui

**Affiliations:** 1 Beijing Institute of Pharmacoloy and Toxicology, Beijing, 100850, People’s Republic of China; 2 School of Medicine, Shanghai Jiaotong University, Shanghai, 200025, People’s Republic of China; 3 Department of Bio-Nano Science and Engineering, Key Laboratory for Thin Film and Microfabrication of Ministry of Education, Research Institute of Micro/Nano Science and Technology, Shanghai Jiao Tong University, Shanghai, 200240, People’s Republic of China

**Keywords:** Ferric ions, Magnetic nanoparticles, Potassium thiocyanate, Mouse tissue, Chemical colorimetric method

## Abstract

This work is aimed to evaluate a method to detect the residual magnetic nanoparticles (MNPs) in animal tissues. Ferric ions released from MNPs through acidification with hydrochloric acid can be measured by complexation with potassium thiocyanate. MNPs in saline could be well detected by this chemical colorimetric method, whereas the detected sensitivity decreased significantly when MNPs were mixed with mouse tissue homogenates. In order to check the MNPs in animal tissues accurately, three improvements have been made. Firstly, proteinase K was used to digest the proteins that might bind with iron, and secondly, ferrosoferric oxide (Fe_3_O_4_) was collected by a magnetic field which could capture MNPs and leave the bio-iron in the supernatant. Finally, the collected MNPs were carbonized in the muffle furnace at 420°C before acidification to ruin the groups that might bind with ferric ions such as porphyrin. Using this method, MNPs in animal tissues could be well measured while avoiding the disturbance of endogenous iron and iron-binding groups.

## Background

Nanotechnology is widely used in drug or gene delivery and targeted therapy [1-4]. The importance of targeted drug delivery is to transport a drug directly to the center of the disease under various conditions and thereby treat it separately, with less effect on other tissues. The nanoparticle designed for drug delivery should be biodegradable and biocompatible [5,6]. Due to its good biodegradability and biocompatibility, the engineered magnetic nanoparticles (MNPs) could be well used in disease diagnosis and even in drug delivery and targeted therapy [7-14]. They can be simultaneously functionalized and guided by a magnetic field [15-17]. The safety of designed MNPs depends on the safety of linked molecules and the magnetic cores. So, evaluating how MNPs distribute and metabolize in different tissues of animals is very important. Moreover, this information is capital to give reference of its optimal dosage and administration route. Magnetic resonance imaging, Prussian blue staining, and transmission electron microscopy were used to detect the distribution of the magnetic nanoparticles *in vivo*. As a vector, MNPs often bond with some conjugate such as cisplatin, and the distribution of conjugates had been used to indicate MNP distribution [18]. Determining iron ions using inductively coupled plasma-mass spectrometry (ICP-MS) is a very sensitive method and has been used to determine the concentration of MNPs in animal tissues [19]. This method could measure the total endogenous and exogenous iron in different tissues of animals. When the tissue contains high concentration of iron ions, the MNP concentration could not be calculated by using this method. Yin et al. have explored the toxicity of Fe_3_O_4_ coated with glutamic acid labeled with Fe^59^ and determined their distribution in mice. Separating from endogenous iron labeled with Fe^59^ could directly catch the trace of MNPs in the different tissues of mice, including the absorption, distribution and clearance, and accumulation in tissues and the probable target organ, and evaluate its pharmacokinetic profile *in vivo*[20]. However, with the same disadvantage of ICP mass, labeled Fe^59^ could not give the information on whether it is a degraded iron ion or an atom in MNPs. Therefore, we try to establish a method to determine the ferric ions in MNPs to observe the metabolism feature of MNPs in animal tissues.

### Experimental materials and methods

#### Animals

CD-1 strain mice were supplied by Vital River Laboratory Animal Technology Co. Ltd. (SCXK(Jing)2006-2009, Beijing, China). They were housed in a controlled environment (21 ± 2°C, 55 ± 5% of humidity, 12-h dark/light cycle with light provided between 6 am and 6 pm). Food and water were given *ad libitum*. All the animal experiments were carried out in the Beijing Center for Drug Safety Evaluation, in accordance with a protocol approved by the Institutional Animal Care and Use Committee of the Center, which is in compliance with the guidelines of the Association for Assessment and Accreditation of Laboratory Animal Care International.

## Materials

The MNPs were from Shanghai Jiaotong Unversity. Iron chloride hexahydrate was from Sinopharm Chemical Reagent Co. Ltd. (Shanghai, China). The MNPs were coated with cetyltrimethyl ammonium bromide at the size of 25 to 35 nm. Magnetic field (MagneSphere Technology Magnetic Separation Stands, Z5341) was from Promega (Madison, WI, USA). Muffle furnace (SX-8-10) was from Tianjin Taisite Instrument Co. Ltd. (Tianjin City, China).

### Establishing a method to determine ferric ions *in vitro*

For quantitive analysis of MNPs the concentration of iron ion was created as a standard for MNPs. Ferric chloride was use to analyze the iron content. Potassium thiocyanide colorimetry showed a good linear relationship and was used to determine ferric ions in MNPs treated with hydrochloride acid at 100°C. Different concentration of 0.5 ml ferric chloride was mixed with an equal volume of 2N hydrochloride acid and boiled for 10 min. The acidified solution was cooled to room temperature and added 120 μl of 5M potassium thiocyanide. Ninety-six-well plates were used in this detection method. The colored reaction product (150 μl/well) was measured at 480 nm on a spectral scanning multimode reader (Varioskan Flash version 2.4.3, Thermo Scientific, Logan, UT, USA). The sonicated and acidified MNPs could be treated in the same way and its ferric ions concentration could be calculated by the ferric chloride standard curve.

### The influence of animal tissue on the determination of ferric ions in MNPs

For the assessment of MNPs in different tissues by colorimetry, the normal mice were narcotized with ether. The whole blood was collected into heparin-coated tube and diluted with nine volumes of saline before mixing with MNPs. Different tissues were harvested. Tissues from three mice were mixed and homogenized in ten volumes of ice-cold saline in a homogenizer (ULTRA-TURRAX T25, IKA-Labortechnik, Staufen, Germany) for 10 s. Three hundred microliters of MNPs at 0.078 to 40 μg/ml in saline was mixed with an equal volume of tissue homogenate and acidified with 80 μl of 6N hydrochloric acid. The acidified solution was centrifuged at 14,000 rpm for 6 min, and then, the supernatants were colorized with 80 μl of 5M potassium thiocyanide.

### The iron background in mouse tissues

One hundred microliters of 10% mouse tissue homogenate was mixed with 500 μl of saline and then treated with proteinase K at the final concentration of 100 μg/ml under 55°C to 65°C for 0.5 h. The samples were then transferred into crucibles, oven dried on a hot plate, and then carbonized in a muffle at 420°C for 2 h. Cooled to room temperature, the samples were acidified with 0.5 ml 1N hydrochloric acid at 100°C for 10 min. Cooled to room temperature, 1N hydrochloric acid was replenished to the final volume of 1.0 ml. Centrifuged at 10,000 rpm for 10 min, 0.5 ml of the supernatants was colorized with 60 μl of 5M potassium thiocyanide. The concentration of ferric ions was calculated referencing the result from ferric chloride standard.

### The determination of MNPs in blood treated with proteinase K with/without magnetic field collection

MNPs at different concentrations in 300 μl of saline were mixed with 30 or 300 μl of mouse whole blood and replenished with saline to the final volume of 600 μl. Aliquots were treated with/without 3 μl of proteinase K, 20 mg/ml, to the final concentration of 100 μg/ml under 55°C to 65°C for 0.5h and centrifuged at 14,000 rpm for 5 min. The 200 μl of the supernatant was mixed with 400 μl of saline to check whether Fe_3_O_4_ remained after centrifugation. The precipitate was washed with 0.6 ml saline, centrifuged two times and suspended in 600 μl of saline. Each sample was added with 80 μl 6N hydrochloric acid and boiled for 10 min. After cooling in a water bath at room temperature, the solutions were colorized with 80 μl 5M KSCN, and their A_480_ were measured.

### Using magnetic field to separate MNPs from the endogenous iron followed by carbonation to ruin ferric ions binding groups

Being a standard, 300 μl of MNPs at 40 μg/ml in saline was diluted with 200 μl of saline, acidified with 500 μl 2N hydrochloric acid, and colorized with 120 μl 5M KSCN. The same aliquots of MNPs, 300 μl in saline, were respectively mixed with 200 μl of saline and 200 μl of saline containing 50% mouse blood. The crucibles containing these solutions were put on a magnetic field, and the MNPs were collected and washed two times with saline. The precipitates could be directly acidified with 1 ml 1N hydrochloric acid or carbonized in a muffle furnace at 420°C for 2 h, cooled to room temperature, and then be acidified. After acidification at 100°C for 10 min and cooling to room temperature, all the samples above were replenished and colorized with 120 μl 5M KSCN. Using the same procedure, MNPs mixed in the mouse tissue homogenate was collected and determined. The collected ratio was calculated and compared with the result of the MNPs in saline.

### Determination of MNPs in the blood of mice treated with MNPs by intravenous injection

CD-1 mice were i.v.-administrated with MNPs at a dose of 7.5 mg/kg. At different time points after MNP treatment, the whole blood was collected in heparin-coated tube. In a crucible, 0.1 ml of whole blood was mixed with 0.9 ml of saline, and MNPs were collected and washed two times with saline accompanied with magnetic field separation. The collected precipitates were carbonized as before. The residues were suspended in 0.5 ml 1N hydrochloric acid and colorized with 60 μl 5M KSCN. The concentration of ferric ions was calculated, referencing with ferric chloride standard with attention on the volume used.

## Results

### The standard of ferric ions determination *in vitro*

**Figure 1:**
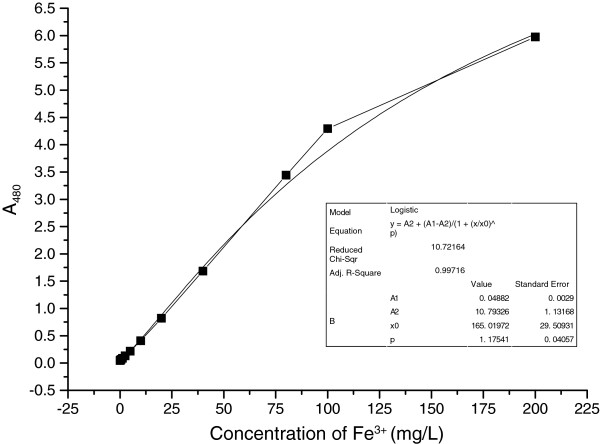
**Standard curve of ferric ions determination.** Different concentrations of ferric ions were colorized with potassium thiocyanate. The value of absorbance at 480 nm was detected. The concentration scale is 0 to 200 mg/L. Each assay was performed in triplicates. Data are presented as means ± standard deviations.

Samples containing different concentrations of ferric ions in water were prepared by diluting the iron chloride solution and acidifying and colorizing following the method described above. The formula was obtained after logistic regression (Figure 1). This examination was repeated three times within the day or within 3 days to calculate the relative standard deviation (RSD) respectively. The RSDs at different concentrations of iron within 1 day or within 3 days were lower than 6% (Table 1).

**Table 1:** The relative standard deviation in the determination of ferric ions (%)

**Ferric ions (mg/L)**	**Within the day**		**RSD (%)**	**Between days**		**RSD (%)**
	**A480**			**A480**		
	**Mean**	**SD**		**Mean**	**SD**	
0	0.0458	0.0027	5.93	0.0462	0.0025	5.32
0.31	0.0540	0.0008	1.48	0.0554	1.90 × 10^−3^	3.43
0.62	0.0665	0.0031	4.68	0.0682	0.0038	5.58
1.25	0.0878	0.0013	1.47	0.0894	2.00 × 10^−3^	2.25
2.5	0.1301	0.0029	2.23	0.1329	0.004	2.97
5	0.2187	0.0049	2.25	0.2253	0.0093	4.13
10	0.4111	0.0084	2.03	0.4219	0.0127	3.02
20	0.8206	0.0297	3.61	0.846	0.0343	4.05
40	1.6834	0.027	1.60	1.6896	0.0202	1.20
80	3.442	0.0633	1.84	3.4985	0.0851	2.43
100	4.2956	0.0671	1.56	4.3911	0.1499	3.41
200	5.9739	0.036	0.60	5.9739	0.036	0.60

The RSD at different concentrations of ferric ions was the relative percentage of the standard deviation with mean value calculated from three determinations of ferric ions in 1 day or 3 days, respectively.

**Figure 2:**
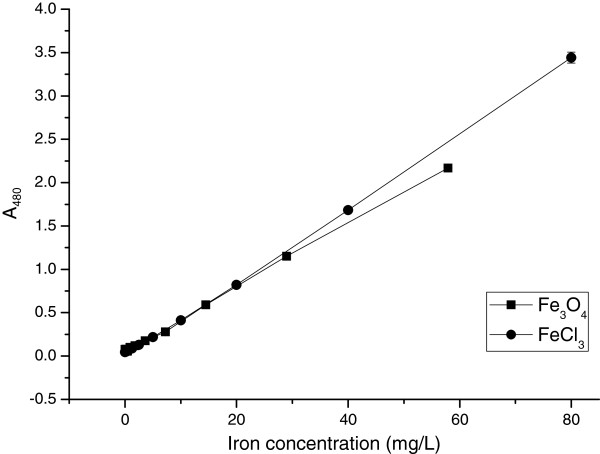
**The determination of iron in magnetic nanoparticles and ferric chloride.** Different concentrations of ferric ions produced from magnetic nanoparticles and ferric chloride were colorized with potassium thiocyanate. Each assay was performed in quadruplicates. Data are presented as means ± standard deviations.

The magnetic nanoparticle sample was diluted according to the ferric ions concentration and acidified and colorized in the same way to measure ferric ions in iron chloride. The values of absorbance at 480 nm of the colorized solution loaded onto the same 96-well plate were determined. The result indicated that this method could be used well to measure the iron concentration and further represent the MNP concentration (Figure 2).

### The influence of blood content on the measure of MNPs

**Figure 3:**
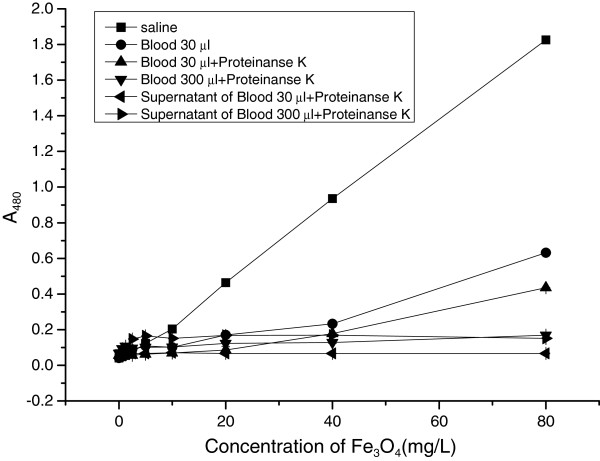
**The ferric ions signal of MNPs in the mouse blood sample.** The influence of blood content on MNP determination was measured by mixed mouse blood and MNPs. Proteinase K used to digest the blood proteins might bind with MNPs or ferric ions. Each assay was performed in triplicates. Data are presented as means ± standard deviations.

In order to elucidate whether the tissue content could disturb the measurement of ferric ions from MNPs, the MNP final concentration at 40 μg/ml of MNP was prepared using saline containing 5% or 50% mouse whole blood. MNP in saline containing 5% or 50% blood was treated with/without proteinase K under 55°C to 65°C and centrifuged. The supernatant and washed precipitate were acidified and colorized respectively. The A480 results indicated that different treatments affected the Fe determination. Even with 5% of whole blood, the A480 decreased significantly, and this decrease could not be alleviated by treating with proteinase K which could digest the protein that might combine with Fe_3_O_4_ or Fe^3+^ (Figure 3). These results reminded that there were some organic components in mouse blood that could catch iron, and these components could be released sufficiently after proteinase K treatment.

### Ferric ion determination after separating from endogenous iron and ruining ferric ion binding groups

The determination of ferric ions in blood indicated that different treatments affected Fe determination. Even with 5% of whole blood, the A480 decreased significantly, and this decrease could not be alleviated by treating with proteinase K which could digest the protein that might combine with Fe_3_O_4_ or Fe^3+^. These results reminded that there were some organic components in mouse blood that could catch iron, and these components could be released sufficiently after proteinase K treatment. Carbonation is one of the methods that could effectively ruin the groups that might bind with iron from MNPs which were separated from endogenous iron existing in the blood under a magnetic field.

**Figure 4:**
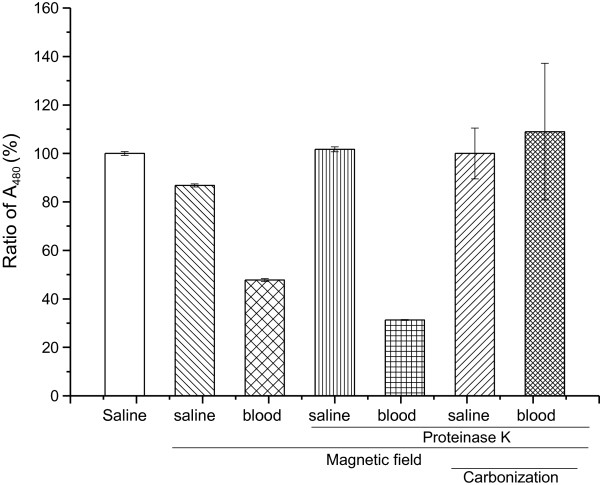
**Determination of MNPs in mouse blood samples by different treatments.** The same amount of MNPs in saline or saline containing 20% of mouse blood was measured respectively. Each assay was performed in triplicates. Data are presented as means ± standard deviations.

The same aliquots of MNPs in saline containing 50% mouse blood were added into crucibles on a magnetic field to collect MNPs and washed two times with saline. The precipitates that were uncarbonized or carbonized in a muffle furnace were acidified and colorized. Compared with the saline standard control, almost 90% of MNPs could be recycled by the magnetic field. When MNPs in saline containing 20% of mouse blood were magnetically recycled and uncarbonized, only half of the ferric ions signal compared with that in saline control was given. Adding proteinase K did not alleviate this decrease. Fortunately, after being digested with proteinase K, collected on a magnetic field, and carbonated in a muffle, the ferric ions signal could almost match the same level of MNPs in the saline control (Figure 4).

**Figure 5:**
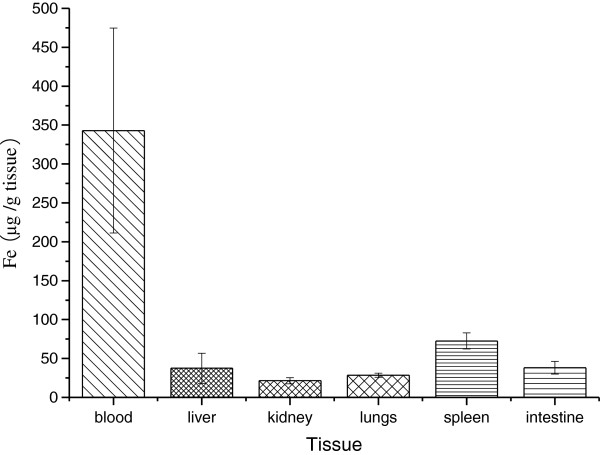
**Endogenous iron in different mouse tissues.** Iron concentrations in mouse blood, liver, kidney, lung, spleen, and intestine were measured. Each assay was performed in triplicates. Data are presented as means ± standard deviations.

**Figure 6:**
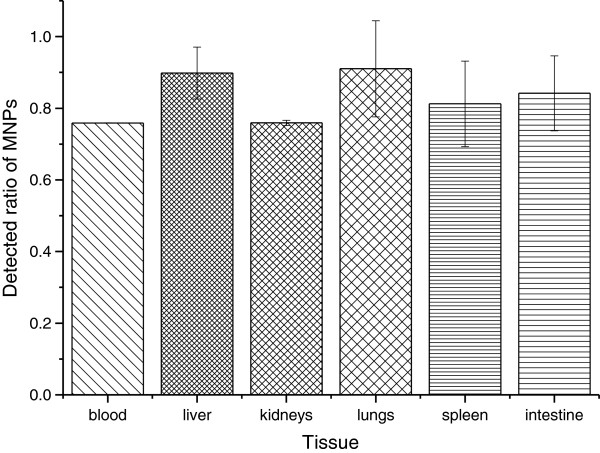
**Determination of MNPs mixed in different mouse tissues.** MNPs were separated from endogenous iron by magnetic field and determined, comparing with the result of MNPs in saline. Each assay was performed in triplicates. Data are presented as means ± standard deviations.

In order to detect how much iron in the different mouse tissues might disturb the MNP determination, the 10% mouse tissue homogenate in saline was prepared. One hundred microliters of tissue homogenate was mixed with 500 μl of saline. After the digestion with proteinase K, carbonation in a muffle, and acidification with HCl, the endogenous ferric ions was determined and calculated referencing to the standard as mentioned before. The results showed that endogenous iron contents were significantly different depending on the kind of tissue. Some tissues such as blood, spleen, and liver have a high concentration of iron, indicating that it is important to separate endogenous iron in MNP detection (Figure 5). Even when the endogenous iron concentration is low, the magnetic collection and carbonation of MNPs are still important because the groups which might bind with ferric ions should be cleared before colorization. Using the same method, the MNPs mixed in different homogenates of mouse tissue were collected under magnetic field after proteinase K digestion and carbonated followed by acidification and colorization. The ratio of ferric ions concentration in different homogenates to that in saline was determined. The result showed that more than 80% of MNPs could be measured without endogenous iron disturbance (Figure 6).

## Discussion

Because nanoparticles (NPs) have the unique physical and chemical properties compared with general substance, their safety could not be well evaluated by normal evaluation methods only. Apart from the toxicity of NPs themselves, the safety of NPs was closely related with the administration way and the distribution path in the animal. Magnetic nanoparticles (MNPs) are drawing increasingly attention due to its unique purposes such as disease diagnosis and drug delivery. Though MNPs could be concentrated at the desired site lead by an external magnet [21] and have been thought to be biodegradable and biocompatible [22], the toxicity of iron in animals should not be ignored because overload of iron oxide has potential toxicity to blood, liver, spleen and kidneys [19,23], especially when the iron was in nanoscale. The limited studies on MNPs toxicity were pointed at the influence of MNP construction, size, surface chemistry and the design of their administration in the toxic outcomes as anticipated [24]. Establishing an experimental method to detect the concentration of MNPs in different tissue and organ of animals is very important to obtain the rule of MNPs distribution and metabolism and then to suspect the potential toxicity.

In this study, chemical colorimetric method to determine the residual MNPs in the tissues of mice has been established. Whole blood was selected as the tissue to establish the MNPs determination method because of the blood half-life is a capital value indicating whether MNPs could evade uptake by RES [25-28]. Compared with other tissues, MNPs in blood was relatively difficult to be measured because the red blood cells contain large amounts of iron. Some research reported that the MNPs in the plasma could be separated with blood cell by centrifugation [29]. It is possible that the MNPs could aggregate and co-precipitate with blood cells. In this research the iron backgrounds in different tissues of mouse were determined elucidating the high bio-iron background in whole blood, spleen, liver and even in intestine. Using ICP mass MNPs, signals in these samples could not be detected accurately especially when lower dose of MNPs was used. So using a magnet to separate MNPs from bio-iron is very important. Ultrasound was used to avoid the aggregation of MNPs and to break the blood cells preventing their precipitation on magnetic field. Proteinase K is effective enzyme to digest proteins might bind with MNPs. Carbonation in muffle and then acidification could oxidize ferrous ions to ferric ions which would be colorized by KSCN. The groups that might complex with ferric ions in tissues could be destroyed when the sample was carbonated. This method will be used to measure the MNPs distribution and metabolism in mice in our further research, especially to validate whether this method could be used to check the MNPs coupled with antibody or other medicine groups.

In this study, we established a method to determine MNPs in mouse tissues without the disturbance of endogenous iron. Even being little tedious, the method is safe and effective. Following more and more uses of MNPs in vivo take place, this method could be of great assistance in measurements of MNPs itself and MNPs coupled with drug or antibody, especially when the tissue containing high level iron and the animal treated with lower dose of MNPs.

## Competing interests

The authors declare that they have no competing interests.

## Authors’ contributions

YW participated in the design of this study and established the method of the MNP determination. WZ determined the influence of tissue contents on the MNP assay. YW carried out the design of this study and participated in the animal experiments. QL, ND, JW, and ZG participated in the tissue separation and homogenation. GG, HH, and KW prepared the MNPs. DC participated in the design and the coordination of this study. All authors read and approved the final manuscript.

## Authors’ information

YW, a senior student at the School of Medicine, Shanghai Jiaotong University, voluntarily worked as a research assistant in the Beijing Institute of Pharmacology and Toxicology during the summer break. YW, is a professor engaged in biochemical pharmacology. JW is a technician engaged in immunological analysis and works with YW. WZ, QL, ND, and ZG were graduate students in the laboratory of YW in the Beijing Institute of Pharmacology and Toxicology. GG, HH, and KW are doing research with DC in the Department of Bio-Nano Science and Engineering, Shanghai Jiaotong University.
